# A Good Exchange

**Published:** 1887-03-19

**Authors:** 


### A GOOD EXCHANGE.

A refuge where those wearied out with pain,
Are taken in and cared for tenderly,
Are helped to win their lost health back again,
By gentle hands that treat them skilfully;
Where kindly ministers and words of cheer,
Give them fresh hope, for dark despair and fear.

Mater.

